# A systematic review of p53 regulation of oxidative stress in skeletal muscle

**DOI:** 10.1080/13510002.2017.1416773

**Published:** 2018-01-03

**Authors:** Kaitlyn Beyfuss, David A. Hood

**Affiliations:** School of Kinesiology and Health Sciences, York University, Toronto, Canada

**Keywords:** Reactive oxygen species, exercise, antioxidant enzymes, mitochondria, apoptosis, diet, chemical agents, transcription

## Abstract

**Background:** p53 is a tumor suppressor protein involved in regulating a wide array of signaling pathways. The role of p53 in the cell is determined by the type of imposed oxidative stress, its intensity and duration. The last decade of research has unravelled a dual nature in the function of p53 in mediating the oxidative stress burden. However, this is dependent on the specific properties of the applied stress and thus requires further analysis.

**Methods:** A systematic review was performed following an electronic search of Pubmed, Google Scholar, and ScienceDirect databases. Articles published in the English language between January 1, 1990 and March 1, 2017 were identified and isolated based on the analysis of p53 in skeletal muscle in both animal and cell culture models.

**Results:** Literature was categorized according to the modality of imposed oxidative stress including exercise, diet modification, exogenous oxidizing agents, tissue manipulation, irradiation, and hypoxia. With low to moderate levels of oxidative stress, p53 is involved in activating pathways that increase time for cell repair, such as cell cycle arrest and autophagy, to enhance cell survival. However, with greater levels of stress intensity and duration, such as with irradiation, hypoxia, and oxidizing agents, the role of p53 switches to facilitate increased cellular stress levels by initiating DNA fragmentation to induce apoptosis, thereby preventing aberrant cell proliferation.

**Conclusion:** Current evidence confirms that p53 acts as a threshold regulator of cellular homeostasis. Therefore, within each modality, the intensity and duration are parameters of the oxidative stressor that must be analyzed to determine the role p53 plays in regulating signaling pathways to maintain cellular health and function in skeletal muscle.

**Abbreviations:** Acadl: acyl-CoA dehydrogenase, long chain; Acadm: acyl-CoA dehydrogenase, C-4 to C-12 straight chain; AIF: apoptosis-inducing factor; Akt: protein kinase B (PKB); AMPK: AMP-activated protein kinase; ATF-4: activating transcription factor 4; ATM: ATM serine/threonine kinase; Bax: BCL2 associated X, apoptosis regulator; Bcl-2: B cell Leukemia/Lymphoma 2 apoptosis regulator; Bhlhe40: basic helix-loop-helix family member e40; BH3: Borane; Bim: bcl-2 interacting mediator of cell death; Bok: Bcl-2 related ovarian killer; COX-IV: cytochrome c oxidase IV; cGMP: Cyclic guanosine monophosphate; c-myc: proto-oncogene protein; Cpt1b: carnitine palmitoyltransferase 1B; Dr5: death receptor 5; eNOS: endothelial nitric oxide synthase; ERK: extracellular regulated MAP kinase; Fas: Fas Cell surface death receptor; FDXR: Ferredoxin Reductase; FOXO3a: forkhead box O3; Gadd45a: growth arrest and DNA damage-inducible 45 alpha; GLS2: glutaminase 2; GLUT 1 and 4: glucose transporter 1(endothelial) and 4 (skeletal muscle); GSH: Glutathione; Hes1: hes family bHLH transcription factor 1; Hey1: hes related family bHLH transcription factor with YRPW motif 1; HIFI-*α*: hypoxia-inducible factor 1, *α*-subunit; HK2: Hexokinase 2; HSP70: Heat Shock Protein 70; H_2_O_2_: Hydrogen Peroxide; Id2: inhibitor of DNA-binding 2; IGF-1-BP3: Insulin-like growth factor binding protein 3; IL-1*β*: Interleukin 1 beta; iNOS: inducible nitric oxide synthase; IRS-1: Insulin receptor substrate 1; JNK: c-Jun N-terminal kinases; LY-83583: 6-anilino-5,8-quinolinedione; inhibitor of soluble guanylate cyclase and of cGMP production; Mdm 2/ 4: Mouse double minute 2 homolog (mouse) Mdm4 (humans); mtDNA: mitochondrial DNA; MURF1: Muscle RING-finger protein-1; MyoD: Myogenic differentiation 1; MyoG: myogenin; Nanog: Nanog homeobox; NF-kB: Nuclear factor-κB; NO: nitric oxide; NoxA: phorbol-12-myristate-13-acetate-induced protein 1 (Pmaip1); NRF-1: nuclear respiratory factor 1; Nrf2: Nuclear factor erythroid 2-related factor 2; P21: Cdkn1a cyclin-dependent kinase inhibitor 1A (P21); P38 MAPK: mitogen-activated protein kinases; p53R2: p53 inducible ribonucleotide reductase gene; P66Shc: src homology 2 domain-containing transforming protein C1; PERP: p53 apoptosis effector related to PMP-22; PGC-1*α*: Peroxisome proliferator-activated receptor gamma coactivator 1-alpha; PGM: phosphoglucomutase; PI3K: Phosphatidylinositol-4,5-bisphosphate 3-kinase; PKC*β*: protein kinase c beta; PTEN: phosphatase and tensin homolog; PTIO: 2-phenyl-4, 4, 5, 5,-tetramethylimidazoline-1-oxyl 3-oxide (PTIO) has been used as a nitric oxide (NO) scavenger; Puma: The p53 upregulated modulator of apoptosis; PW1: paternally expressed 3 (Peg3); RNS: Reactive nitrogen species; SIRT1: sirtuin 1; SCO2: cytochrome c oxidase assembly protein; SOD2: superoxide dismutase 2; Tfam: transcription factor A mitochondrial; TIGAR: Trp53 induced glycolysis repulatory phosphatase; TNF-a: tumor necrosis factor a; TRAF2: TNF receptor associated factor 2; TRAIL: type II transmembrane protein.

## Introduction

Skeletal muscle is a highly malleable tissue, capable of conforming to numerous metabolic and physiological requirements imposed by various stressors. This adaptable property of skeletal muscle is a trait that is essential for regulating cellular homeostasis in response to various stressor intensities and durations. As skeletal muscle comprises approximately 40% of the total body mass, maintaining muscle health and function is imperative to sustaining whole body health and protection against chronic disease [1]. Dysfunctional skeletal muscle, as seen with chronic inactivity, aging, and tissue damage, is a precipitating cause towards conditions such as type II diabetes, cardiovascular disease, and cancer [2–4]. Depending on the imposed stress, various signaling pathways may be activated and/or repressed to ensure that skeletal muscle health is preserved. Therefore, it is essential to understand the molecular mechanisms involved in the maintenance of skeletal muscle health under the demand of various oxidative stressors to ultimately determine future therapeutic modalities.

Oxidative stress is a result of the imbalance between the induction of reactive oxygen species (ROS) and the cells ability to metabolize them [5,6]. Many molecular signaling events characterize oxidative stress, including the production of hydrogen peroxide, superoxide, and peroxynitrite [6–8]. When produced in high quantities, they place a high demand on the detoxification systems, and lead to the disruption of numerous metabolic pathways causing upwards of 200 human diseases [7].

p53 is a well-known tumor suppressor protein and is commonly referred to as the ‘Guardian of the Genome’ for its role as a major determinant of cell fate. p53 regulates the expression of an assortment of genes involved in maintaining homeostasis, including those involved in cell cycle regulation, redox homeostasis (i.e. antioxidant enzyme production), DNA replication and repair, apoptosis, and autophagy [9–13]. Various forms of oxidative stress lead to post-translational modifications of p53, allowing it to regulate genes to cause either beneficial outcomes, such as the upregulation of mitochondrial biogenesis, or more dysfunctional consequences such as cellular senescence and apoptosis [6,13–17]. The divergent effects of p53 likely depend on the degree of oxidative stress imposed. As such, delineating the role of p53 in response to various intensities and durations of oxidative stress and its ensuing regulation of skeletal muscle health is essential to determine the molecular signaling pathways involved in maintaining cellular homeostasis.

With this purpose, we set out to analyze various modalities of imposed oxidative stress and the subsequent signaling pathways activated in skeletal muscle, with a specific focus on the role of p53 in regulating these pathways. This review will describe the signaling mechanisms controlled by various stressors and how, under these influences, p53 ultimately functions to maintain muscle health and function, while additionally focusing on the pathways that lead to pathological consequences.

## Methods

A systematic review of the experimental evidence was performed on the role of p53 in regulating oxidative stress in skeletal muscle. Articles published in the English language were evaluated through Pubmed, ScienceDirect, and Google Scholar search engines using the key terms ‘p53’ and ‘Skeletal Muscle’ and ‘Oxidative Stress’, while restricting against ‘Cancer’ and ‘Tumor’. Primary research studies included for comparison involve only animal and cell culture models. Important studies involving human subjects published in this area are discussed where applicable, but not compiled in the data tables for analysis in order to keep the review focused. However, a list of excluded human studies can be found in the appendix. All materials published between January 1, 1990 and March 1, 2017 were included for review.

**Figure 1.**
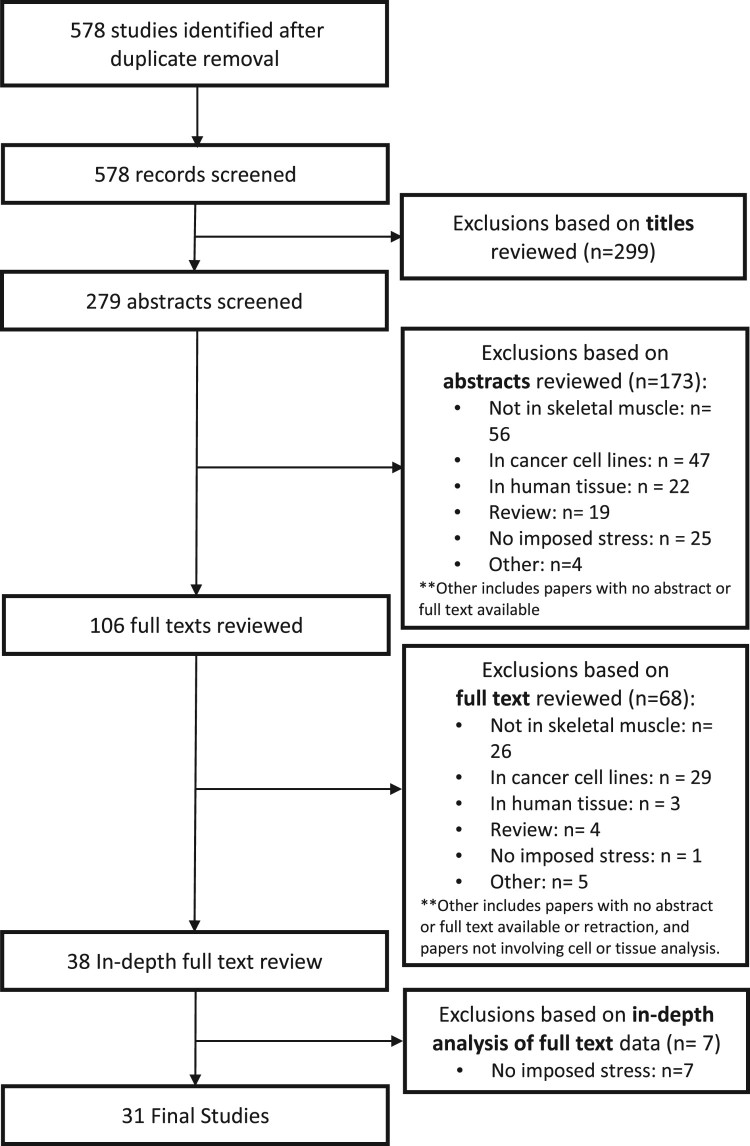
Layout of systematic analysis for literature inclusion. Various databases were utilized to identify all studies published between 1990 and 2017 in which an animal or cell culture models under imposed oxidative stress were assessed to examine the effects on skeletal muscle and downstream proteomic markers. We screened 578 articles published in the English language; 38 were eligible for critical appraisal resulting in a total of 31 articles to be included in this review.

Studies were initially screened based on title; duplicate articles and articles not publishing original research were excluded. Studies were then screened by a review of abstracts fitting the appropriate inclusion criteria (Table 1). If abstracts fit the criteria, a complete full-text analysis was performed using similar inclusion criteria. A total of 578 studies were included for review, and following exclusion, 31 studies remained for further analysis (Figure 1).

**Table 1.** Inclusion and exclusion criteria. At the various stages of study analysis for inclusion in review, papers were included and excluded based on the above criteria. At the point of study inclusion, some papers satisfied the requirement of more than one form of oxidative stress.

Stage	Stage description	Inclusion criteria	Exclusion criteria
1	Journal title analysis	Key words: p53, skeletal muscle Form of oxidative stress	Key words: Human, cancer/tumour/adenoma/sarcoma, review, tissues (other than skeletal muscle) Different language Methodology paper
2	Abstract analysis	p53 signaling activation Imposed oxidative stress Skeletal muscle tissue Animal or cell model	Tissues/cells (other than skeletal muscle or myoblasts) Human Model Cancer cells/ tissues infected with cancer Review or methods paper No abstract available (retraction of paper)
3	Full text analysis	p53 signaling activation Imposed oxidative stress Skeletal muscle tissue Animal or cell model Oxidative stress (exercise, tissue manipulation, chemical and medicinal agents, diet modification, hypoxia, irradiation)	Full text is unavailable No form of imposed oxidative stress

Studies were organized based on the oxidative stress intervention and categorized into one of six groups: exercise, diet modification, tissue manipulation, chemical and medicinal agents, irradiation, and oxygen deprivation. A limited number of studies fulfilled more than one category. Data extraction was performed to assess study methodology, the effect of upstream regulators and downstream targets of p53-regulated signaling pathways affected by the oxidative stress, and the application and relevance of the model. Qualitative analyses were performed to compare within and between interventions on the effectiveness of the model based on the induction of oxidative stress, and the role of p53 and other signaling mechanisms in regulating stress intensity and duration. A meta-analysis was not performed on this data.

## Results

### The role of p53 in mediating oxidative stress imposed by acute and chronic exercise

Acute and chronic contractile activity triggers a plethora of signals that induce beneficial metabolic and biochemical adaptations to enhance muscle health and performance. For example, the moderate increases in ROS produced by exercise are able to repair and strengthen the oxidative capacity of the cell by increasing mitochondrial content and fuel oxidation, and thus has dissimilar effects compared to the consequences of chronic, high levels of ROS [18–20]. In this review, a total of seven exercise studies (two acute, five chronic) have been analyzed to establish the role of p53 in regulating this physiologically beneficial form of oxidative stress to augment muscle health (Table 2).

**Table 2.** Literature compiled on oxidative stress imposed through various exercise modalities.

Publication information (Ref#)	Research model	Age at start (weeks)	Stress intensity	Length of stress	p53 expression (residue modification)	p53 cellular localization	Regulation of signaling pathways with stress		Signaling pathway(s) activated	Path model
							mRNA	Protein		
Acute exercise programs										
Saleem, Hood, J Physiol, 2013 [24]	(C57Bl/6J p53 KO and WT mice *n* = 6	12	15 m/min for 90 min (±3 h recovery)	90 min	↓ p53 mRNA (w/acute and acute + recovery)	↑ p-p53 @ ser^15^ in SS and IMF mito ↓ p53 in nucleus	↑ PGC-1*α*, ↑Tfam, ↑NRF-1 ↑COX-IV, ↑CS, ↑COX-I greater with 3 h recovery	n/a	Mitochondrial biogenesis, mtDNA integrity (tfam-p53)	n/a
Saleem et al., Am J Physiol Cell Physiol, 2014 [12]	C57BL/6J p53 KO and WT mice *n* = 6	8	15 m/min for 90 min (±3 h recovery)	90 min	n/a	n/a	↑ PGC-1*α*, ↑Tfam, ↑NRF-1, ↑COX-IV greater with 3 h recovery	↑ p-p38 MAPK@ tyr^180^, ↑p-AMPK @ tyr^172^, ↑p-CaMKII @ tyr^286^, ↑LC3-II (mito)	Mitochondrial biogenesis, autophagy	n/a
Chronic exercise programs										
Qi et al., Free Radic Biol Med, 2011 [18]	Goto-Kakizaki rats *n* = 7	10–12	Progressive: 20 m/min @ 30 min → 1 h	8 weeks (6 days/week)	↓ p53 protein	n/a	↓TIGAR, ↑ mtDNA (ATPase + CytB)	↓ TIGAR, ↑ COX II, ↑ GSH, ↑ GSH:GSSG	Insulin resistance, glycolysis, mitochondrial biogenesis	Non-obese type II diabetes
Safdar et al., Skelet Muscle, 2016 [32]	C57Bl/6J PolG WT and KO, p53 MSKO, and PolG-p53 MSKO mice *n* = 4–6	12	15 m/min @ 45 min	26 weeks (3 days/week)	No change	↓ Nuclear p53 ↑mito p53 (↑ p53-polG-tfam @ Cox II + cytob)	↑ mtDNA copy number	↑ SOD2, ↑ catalase, ↑ Tfam, ↑ ERR*α*	Antioxidant, mitochondrial biogenesis	n/a
Park et al., Circ Res, 2009 [26]	C57BL/6J p53 KO and WT and HT mice *n* = 5	8–12	Progressive: 10 m/min @ 40 min → 14 m/min @ 90 min)	5 weeks (5 days/week)	n/a	n/a	↑ Tfam, ↑ mtDNA copy number	↑ SDH	Mitochondrial biogenesis, glycolytic vs. oxidative respiration	n/a
Saleem et al., Physiol Genomics, 2009 [11]	C57BI/6J p53 KO and WT mice *n* = 7–8	9–12	Voluntary wheel running program; acute in situ stimulation (1 and 3 TPS)	6–8 weeks	↑ p-p53 @ ser^15^ (acute)	n/a	n/a	↑ COX (chronic), ↑ p-AMPK @ tyr^172^ + ↑ p-p38 @ tyr^180^ (acute)	Mitochondrial biogenesis	n/a
Siu, Always, J Appl Physiol, 2005 [33]	(Japanese Coturnix quails *n* = 8	8 and 208	Stretch-overload model (12% body weight over the left humeral-ulnar joint for joint extension)	7 and 21 days	n/a	↑ Nuclear p53	n/a	↑ Id2 (nuclear) (7 days in PAT; 7 and 21 days in ALD)	Hypertrophy	n/a

Notes: Literature (seven studies) was grouped by acute (two studies) or chronic (five studies) exercise. Arrows indicate whether markers regulated by this form of stress increased (↑) or decreased (↓) in expression and/or activation. If a WT vs. KO model is employed, only the results from the WT group are indicated. A caveat to this is the study published by Safdar et al., Skelet Muscle, 2016 which examines exercise in Polg1 MSKO models only. Various time measurements are indicated for the length of imposed stress. WT: wildtype; KO: knockout; HT: heterozygous; MSKO: muscle-specific KO; mito: mitochondria; SS: subsarcolemmal; IMF: intermyofibrillar; PAT: patagialis; ADL: anterior latissimus dorsi.

One bout of acute exercise is sufficient to initiate transcriptional signaling towards mitochondrial biogenesis, and thus ultimately improves the oxidative capacity of skeletal muscle with the assistance of p53 [21–23]. Increased activity of upstream kinases such as AMPK and p38 MAPK leads to Ser^15^ phosphorylation of p53, allowing for both nuclear and mitochondrial localization [12,22,24]. In the nucleus, p53 binds to response elements within the promoter of PGC-1*α* leading to the expression of nuclear genes encoding mitochondrial proteins (NUGEMPs) such as Tfam, COX-IV, SCO2, and AIF [12,17,22–26]. A recovery period of ∼3 h further improves signaling events (i.e. nuclear p53 localization and transcriptional regulation) to enhance mitochondrial biogenesis marker expression in both rodent and human models [24,27,28]. p53 localization to mitochondria enhances mitochondrial biogenesis in both subsarcolemmal and intermyofibrillar populations through its exonuclease activity on mtDNA, and by increasing the expression and binding of Tfam and Polg1 to mtDNA to maintain genomic integrity [12,22,24,29–31].

In contrast to acute exercise, training is classified as repeated bouts of endurance exercise interspersed with recovery sessions over a period of time. The result of chronic exercise is a heightened adaptive state in which the signaling response to each exercise bout is attenuated, including reduced ROS production [3]. This adaptation consists of increased mitochondrial content and improved mtDNA integrity, reduced telomere shortening and consequentially decreased cellular senescent and apoptotic signaling, less emphasis on glycolytic energy utilization and more on oxidative phosphorylation, reduced lactate production, and ultimately improved VO_2_ max and skeletal muscle performance [11,18,26,32,33]. These beneficial adaptations, observed through the implementation of knockout (KO) models, are dependent on increased p53 nuclear and mitochondrial abundance under variable chronic exercise regimens. Despite the fact that these beneficial alterations in cellular milieu can be optimized to enhance skeletal muscle health based on p53 signaling activation and localization, the absence of p53 does not seem to necessarily hinder the ability to adapt with exercise [11,18,33]. Though there is a reduced exercise capacity in p53 knockout mice, there is a similar increase in mitochondrial content compared to wildtype (WT) mice, indicating that exercise provokes the overlapping of redundant signals to ultimately induce the observed adaptations in mitochondria with training [11].

The subcellular localization of p53 is indispensable for exercise-mediated mtDNA repair and mitochondrial biogenesis, notably through the initiation of the Tfam-Polg1 complex in the mitochondria, independent of the nuclear regulation of TIGAR, SCO2, p53R2, and FDXR expression [26,32]. An interesting controversy arises, however, with human models wherein short interval training (SIE), more than continuous exercise (CE), increases p53 Ser^15^ phosphorylation and PGC-1*α* nuclear localization [27]. Indeed, higher training volumes result in increased p53 content, as well as mitochondrial biogenesis and augmented respiration [34]. However, another study reported no differences in signaling or gene expression responses when high and lower intensity exercise regimens were performed once matched for workload [35]. Therefore, further examination into the variable parameters associated with exercise, including sex, age, exercise protocol, and muscle type is required.

### The role of p53 in mediating oxidative stress imposed by diet modification

There is ample evidence confirming that the modification of nutritional resources can alter physiological equilibria leading to specific muscle adaptations. As such, diet modification is considered a cellular stressor through its disruption of homeostasis. p53 is an essential upstream regulator of metabolism whereby it specifically transcribes TIGAR, SCO2, HK2, GLS2, and GLUT 1 and 4 genes [36,37]. Upregulation of these genes enhances oxidative phosphorylation for energy production and reduces the emphasis on glycolysis in order to prevent the Warburg effect, a hallmark of cancer metabolism [36,37]. In this review, a total of six studies (two diet modification, two caloric restriction, and two fasting) were analyzed to determine the signaling mechanisms involved with diet modification and how they may be regulated by p53 (Table 3).

**Table 3.** Literature compiled on oxidative stress imposed through various dietary modifications.

Publication information (Ref#)	Research model	Age at start (weeks)	Stress intensity	Length of stress	p53 expression (residue modification)	p53 cellular localization	Regulation of signaling pathways with stress		Signaling pathway(s) activated	Path model
							mRNA	Protein		
Diet modification										
Nakahara et al., Am J Physiol Endocrinol Metab, 2003 [62]	Wistar rats *n* = 8–10	6–7	(1) Chronic alcohol (35% total dietary energy) + restriction (2) Acute alcohol (75 mmol/kg) + starvation	(1) 6–7 weeks alcohol (2) 24–48 h starvation + 2.5 h alcohol	1) n/a 2) ↓ p53 mRNA (starvation)	n/a	(1) ↑ c-myc (F + alcohol), (2)↑ c-myc (alcohol and/or starvation)	n/a	Proto-oncogenic activation, pre-apoptotic effect	Chronic alcohol exposure
Yokoyama et al., Cell Rep, 2014 [56]	C57BL/6 Tie2-Cre w/ p53, p53 + eNOS, Mdm4 mice *n*= 5–10	4	HF/HS	8 weeks	↑ p53 protein	n/a	↑Cdh5, ↑ Kdr, ↓ mtDNA content	↑ Insulin-induced p-Akt @ ser^473^, ↓p-eNOS @ser^1177^, ↓ PGC-1*α*, ↓ Akt, ↑PHLDA3, ↓GLUT1	Endothelial cell expression, glucose metabolism, mitochondrial biogenesis	Insulin resistance + obesity
Dietary restriction										
Edwards et al., BMC Genomics, 2007 [44]	C57BL/6NHsd mice *n* = 5	6–7	26% <normal diet (∼98 kcal/week)	130 weeks	↓ p53 mRNA	n/a	↓ MYOD1, ↓PLAGL1, ↓p21, ↓IGF-BP3, ↓Krt15 mcII, ↓ PERP, ↓sestrin 1, ↓PTEN, ↓ PEG3, ↓ RB1, ↑Bcl6 B cell leukemia, ↑ cyclin G1, ↑ mdm2, ↓ bbc3/puma, ↓ pmaip1/noxa, ↓tnfrsf10b/ killer/dr5, ↓bok	↓ p21, ↓ Gadd45a	cellular senescence, apoptosis *77% prevention by CR on p53-related genes	n/a
Assaily et al., Mol Cell, 2011 [49]	C2C12 myoblasts w/Lpin1, p53 KO	n/a	1 or 0 mM glucose + 24 h fasting	2 h	↑p53 and p-p53 @ ser^18^	↑53BP1 nuclear	↑ Lpin1	↑ p-AMPK @ Thr^172^, ↑ p-ACC @ ser^79^, ↑p-ATM @ ser^1987^, ↑ p-H2AX	Glycolysis, pentose phosphate pathway, fatty acid oxidation	n/a
Fasting/complete food withdrawal										
Schupp et al., BMC Genomics, 2013 [38]	C57B1/6J mice *n* = 25	10–12	`Withdrawn food	Timecourse: 0, 3, 6, 12, 24, 48 h	↑ p53 mRNA	n/a	↑ Pkc1 (3–48 h), ↑ G6P (3–20 h), ↑ Pcx (48 h), ↑Gyk (12–24 h), ↑Hmgcs2 (12–48 h), ↑ Fgf21 (24 h), ↑ Ppargc1a (24–48 h), ↑Ppara (3 + 24 h), ↑ Cdkn1a, ↑Ses1 + 2, ↑ Lpin1, ↓ Srebf1 + 2, ↓ Acss2, ↓ Acaca, ↓ Fasn, ↓ Scd1 + 2, ↑ Ddit4 (12–48 h) * review study for full list	↑ AMPK, ↓ mTORC1, ↓ Ppargc1a	Fatty acid oxidation, cholesterol biosynthesis	n/a
Aquilano et al., Antioxid Redox Signal, 2013 [48]	C57/BL/6J and CDI mice *n* = 4	5	Withdrawn food	24 h	↑ p53 protein	↑ p53 nuclear + ↑ binding PGC-1*α* @ −2317	↑ PGC-1*α*, ↑ SOD2	↓ GSH, ↑ PGC-1*α*, ↑SOD2	Antioxidant	n/a

Notes: Literature (six studies) was grouped by diet modification (two studies), dietary restriction (two studies), or fasting/complete food withdrawal (two studies). Arrows indicate whether markers regulated by this form of stress increased (↑) or decreased (↓) in expression and/or activation. If a WT vs. KO model is employed, only the results from the WT group are indicated. Various time measurements are indicated for the length of imposed stress. KO: knockout; Cre: creatine; HF/HS: high fat/high sucrose; F: fasting; CR: caloric restriction.

Caloric restriction extends longevity by reducing metabolic risk factors including blood pressure, serum fasting glucose, and total cholesterol [38–40]. Furthermore, in humans, reduced carbohydrate/glycogen availability, with the addition of exercise, maximizes beneficial adaptations by inducing a glycogen-mediated effect on p53 signaling leading to increased transcription of mitochondrial biogenesis markers [41–43]. When deprived of dietary nutrients, the body derives glucose from liver glycogen stores which quickly deplete, causing a metabolic switch in peripheral organs such as muscle to utilize fatty acids from adipose tissue as their primary energy source [38,44–46]. This switch in metabolic fuel usage is mediated by numerous upstream regulators, one of which is p53. The upregulation of p53 in response to fasting-induced oxidative stress enhances both antioxidant production and fatty acid oxidation through the specific mechanisms detailed below.

Glucose withdrawal upregulates nitric oxide production and activates ATM kinase through Ser^1987^ phosphorylation [47–49]. This initiates the cellular stress-response pathway by Ser^18^ phosphorylation of p53 leading to increased nuclear localization [38,49]. When p53 is bound to PGC-1*α* in the nucleus, it can control Lipin-1 expression, a regulator of fatty acid metabolism, to increase fatty acid oxidation (FAO) and triglyceride synthesis, as well as upregulate TIGAR to reduce the emphasis on glycolytic energy production [38,49–52]. p53 can also upregulate GLS2 expression to catalyze the conversion of glutamine to glutamate and increase *α*-ketoglutarate to enhance mitochondrial respiration and ATP production [49,53]. This occurs concomitantly with an enhancement in antioxidant defences through the upregulation of GSH content which directly (reacts with ${\text{O}}_{2}^{-}$) and indirectly (revitalizing antioxidants) scavenges ROS [49,53,54]. In addition to the regulation of metabolic fuel pathways, p53 also controls specific cell signaling pathways. When in the nucleus, phosphorylated p53 can interact with PGC-1*α* to enhance Nrf2 and SOD2 antioxidant expression, concomitant with sestrin activation, to buffer the harmful effects of ROS/RNS to protect the cell during moderate to prolonged fasting states [48,49]. Furthermore, with fasting there is an upregulation of p21 and Gadd45a cellular senescent regulators, and a decrease in apoptotic mRNA such as bok, puma, noxa, and dr5 [44]. Therefore, during fasting or under reduced glucose conditions, p53 leads to an upregulation of antioxidant production, cellular senescence, and FAO, while reducing glycolytic and apoptotic signaling.

On the other hand, the effect of chronic supplementation of metabolic fuels in excess to a normal diet enhances vascular oxidative stress leading to the development of pathological conditions including obesity, diabetes, and coronary artery disease [55,56]. With elevated glucose and fatty acid consumption, there is an increase in endothelial cell p53 expression via reduced Mdm4 negative regulation of p53 [56,57]. p53 accumulation can prevent the phosphorylation of eNOS via Akt downregulation by PTEN, while also reducing the expression of PGC-1*α* and its downstream targets NRF-1 and Tfam, which are involved in vascular angiogenesis and mitochondrial biogenesis, respectively [56,57]. Reductions in additional PGC-1*α*-target genes involved in FAO including Acadl, Acadm, and Cpt1b can lead to fat accumulation [58]. p53 upregulation can also reduce insulin sensitivity by downregulating endothelial GLUT1 through direct transcriptional repression, causing impaired glucose uptake which can lead to oxidative stress by preventing substrate availability for energy production [56,59–61]. Interestingly, the deletion of endothelial p53 inhibits the diet-induced downregulation of GLUT1 expression in these cells to improve glucose uptake into skeletal muscle [56]. In addition to reducing GLUT1 expression, p53 has an inhibitory effect on the GLUT4 promoter within skeletal muscle, suggesting that p53 can negatively regulate insulin sensitivity in this tissue and induce insulin resistance [59]. However, with malignancy-induced mutations in the p53 DNA-binding domain, p53 repression on the GLUT1 and 4 promoters is lifted leading to enhanced insulin sensitivity and glucose tolerance to facilitate the Warburg effect for tumorigenesis [37,59].

Another dietary modification includes the addition of toxic substances into the diet, such as alcohol in excessive quantities, which can lead to reduced contractile proteins (myosin, desmin, actin, troponin) in the muscle itself causing alcoholic myopathy, defined by cramps, impaired muscle strength, difficulties in gait, and reduced lean tissue mass [62–64]. Interestingly, the expected increase in the expression of p53 and Bcl-2-family proteins with increasing ethanol dosage does not occur. Instead, there is an activation of the proto-oncogene c-myc by acetaldehyde, specifically leading to HSP70 binding and initiation of the unfolded protein response and apoptotic cellular stress pathways, either dependent or independent of p53 [62,65,66]. Excess consumption of almost any macronutrient can cause debilitating consequences, and therefore illuminating the toxic pathways activated in mediating this oxidative stress, as well as those that may combat it, are essential to developing therapeutic modalities for these diet-induced pathologies.

### The role of p53 in mediating oxidative stress imposed by pathological tissue manipulation

In contrast to the effect of healthy physical activity on skeletal muscle, this tissue is also subject to reduced activity levels brought about by trauma, denervation, or immobilization. With the knowledge that p53 plays a role in regulating overall cellular homeostasis, the intensity of the various physical manipulations of this kind may lead to alternate functions required of p53 to mediate or attenuate this stress. A total of five studies have been selected to assess the potential variations in p53-mediated regulation of muscle adaptations under these conditions (Table 4).

**Table 4.** Literature compiled on oxidative stress imposed through various modalities of tissue manipulation.

Publication information (Ref#)	Research model	Age at start (weeks)	Stress intensity	Length of stress	p53 expression (residue modification)	p53 cellular localization	Regulation of signaling pathways with stress		Signaling pathway(s) activated	Path model
							mRNA	Protein		
Morimoto et al., J Atheroscler Thromb, 2011 [67]	KK/Ay and C57Bl/6J mice *n* = 8	9	Unilateral hindlimb ischemia surgery	2 weeks	↑ p53 protein	n/a	n/a	n/a	n/a	Type II diabetes
Schwarzkopf et al., Genes Dev, 2006 [68]	BALB/c + C57BL/6 p53 KO and WT mice *n* = 6-7	7	C26 tumor graft and tissue crush injury	3 weeks	↑ p53protein	n/a	↑ Atrogin-1	↑ PWI	fiber regeneration, atrophy	cachexia, cancer
Fox et al., Am J Physiol Endocrinol Metab, 2014 [69]	C57B1/6J p53 MSKO, ATF4 MSKO, and p53/ATF4 MSKO mice *n* = 5–6	8–12	Unilateral hindlimb immobilization (ankle joint + TA)	Timecourse: 1–3 days	↑ p53 protein (1–3 days)	n/a	↑ Gadd45a, ↑ histone deacetylase 4, ↑muscle RING finger, ↑ muscle atrophy F-box/atrogin-1, ↑ p21, ↑1-subunit of the nicotinic acetylcholine receptor, ↑ ATF4	↑ p21	Atrophy, cellular senescence	Immobility
White et al., Int J Dev Biol, 2002 [70]	C57BL/6J p53 KO and WT mice *n* = 10	2–32	Autographs of EDL over TA muscles and tissue crush injury	2–14 days grafting; 3, 5, 7, 10, 14 days grafting and 7 and 14 days crush injury	n/a	n/a	n/a	↑ Desmin (day 5)	regeneration, atrophy	muscle injury
Nakazawa et al., PLOS ONE, 2017 [71]	C57BL/6 and iNOS KO mice *n* = 3–5	8	Abdominal full-thickness burn injury (30% of total body surface area)	6 s abdominal immersion and both sides of the flank for 4 s in 80˚C water; Timecourse: @ 0, 1, 3, 7 days	↑acetyl – p53 @ Lys^379^ (1-3 days) ↑p-p53 @ Ser^15^ (3 days)	n/a	↑ 1L-1*β*, ↑TNF-*α*, ↑IFN-*γ*, ↑ TRL-4, ↑ Bax, ↑ FasL (3 days) ↑ Murf1, ↑atrogin-1 (3 days)	↑ iNOS (3 days), ↑acetyl – p65 @ Lys ^310^ (3 days), ↑p-p65 @S^276^ + S^311^ (3 days), ↓ eNOS (3 days), ↑ S-NO SIRT1 (3 days), ↑ caspase 3 (3 days), ↑HMGB1 (3 days)	Atrophy, inflammation, apoptosis	Burn

Notes: Literature (five studies) on various modalities was not grouped as each model was very different. Arrows indicate whether markers regulated by this form of stress increased (↑) or decreased (↓) in expression and/or activation. If a WT vs. KO model is employed, only the results from the WT group are indicated. Various time measurements are indicated for the length of imposed stress. WT: wildtype; KO: knockout; MSKO: muscle-specific KO; EDL: extensor digitorum longus; TA: tibialis anterior.

The most common manipulations of muscle include crush injury, tissue autografting, disease, and burn injury which can lead to direct skeletal muscle atrophy, or indirect atrophy through immobilization [67–72]. Tissue crush injury, involving a significant disruption to muscle structure but preserving blood and nerve supply, leads to impaired myotube formation, a greater percentage of necrotic tissue, and increased desmin protein which is exaggerated in the absence of p53 [68,70]. However, p53 does not seem to be required for muscle regeneration following injury since its other family members, p63 and p73, compensate for its loss [70]. Major trauma or full-thickness burn injury can induce significant metabolic dysfunction (hyperglycemia, insulin resistance, lactate production) which contributes to inflammation, increased catabolism, and ultimately muscle wasting [71,73]. Though p53 is known to regulate the inflammatory response and apoptosis, its role in mediating the cellular responses to major trauma has not yet been examined. One study revealed that three days-post burn injury increased the cytokine-mediated activation of iNOS to produce NO as a defence mechanism, leading to the inactivation of SIRT1 and the subsequent activation of p65 NF-kB and p53 [71]. This activation initiates the inflammatory response by increasing TNF-*α* and IL-1*β* expression, while concomitantly increasing insulin resistance and apoptosis respectively, to induce muscle wasting [71,74]. Another important and more common cause of muscle atrophy is immobilization. The immobilization-induced increase in p53 allows it to function as a key ATF-4-independent mediator of muscle atrophy, leading to direct p21 activation and subsequent tissue atrophy of all fiber types through cell cycle-dependent mechanisms [68]. Additionally, results from hindlimb suspension models of inactivity have shown increased cytosolic Id2 protein and p53 levels, which play a role in apoptosis-related atrophy [74,75]. Interestingly, atrophic stimuli affect skeletal muscle fiber types differently, with cachexia-associated atrophy leading to a greater reduction in slow fiber type size, while fast fiber types are more susceptible to atrophy when p53 is removed, possibly through the upregulation of the atrogin-1 ubiquitin ligase-mediated pathway [68,74].

Additional roles for p53 can be observed in disease settings that induce pathological muscle degeneration, such as seen with diabetes and cancer cachexia. A precursor to many diseases, diabetes leads to cellular stress by reducing proteasomal degradation of p53 through the inhibition of the negative Mdm2/Akt upstream regulatory pathway, leading to an abnormal accumulation of p53 and the induction of apoptosis [67]. Interestingly, increased p53 expression in adipose tissue appears to assist in reducing adiponectin levels, causing the development of insulin resistance and type II diabetes [67,76]. On the other hand, with cancer cachexia, TNF-*α*-mediated inhibition of myogenin and MyoD, and activation of p53-dependent cell death proteins, leads to the inhibition of myogenic differentiation via upregulation of atrogin-1 to enhance apoptosis and atrophy [68,77,78]. Therefore, understanding the global role of p53 within the organism and how it contributes to muscle wasting with pathological tissue manipulations such as these may lead to the identification of more effective therapeutics. Treatment modalities such as statins (atorvastatin) for cardiovascular disease and diabetes, or progestagens for cancer cachexia, in addition to physical rehabilitation during hospitalization, can ultimately function to reduce muscle wasting symptomatology and associated comorbidities [67,79].

### The role of p53 in mediating oxidative stress imposed by oxygen deprivation

Cellular respiration, an essential metabolic process for energy generation, requires oxygen as the terminal electron acceptor in oxidative phosphorylation to produce ATP energy. Low oxygen tension denotes a hypoxic microenvironment which may be induced by mechanisms such as ischemia or high-altitude exposure, causing detrimental physiological consequences [80–82]. p53, a regulator of mitochondrial metabolism, is suspected to play a major role in compensating for this insult and may be able to provide protective adjustments against cellular damage. We examined two studies that were designed to elucidate the role of p53 in regulating the cellular responses to hypoxic stress (Table 5).

**Table 5.** Literature compiled on oxidative stress imposed through oxygen deprivation.

Publication information (Ref #)	Research model	Age at start (weeks)	Stress intensity	Length of stress	p53 expression (residue modification)	p53 cellular localization	Regulation of signaling pathways with stress		Signaling pathway(s) activated	Path model
							mRNA	Protein		
Wang et al., J Biol Chem, 2015 [80]	Primary myoblasts	n/a	Hypoxia (1% O_2_)	2.5 min each day for 2 days	↑ p53 mRNA and protein	n/a	↑ Bhlhe40, ↑ Bhlhe41	↑ Bhlhe40, ↓ MF20+, ↓ Myog	Cell cycle (H1F1*α*), glycolysis, differentiation	Hypoxia
Zhang et al., Gene,2013 [84]	T.S. Elegans *n* = 4	Adult	Hypoxia (dechlorinated water bubbled with N_2_ gas for 1 h)	5 or 20 h	↑ p53 protein (20 h) ↑ p-p53 @ ser^37^, ser^46^, ser^392^ (5 h) and @ ser^6^, ser^9^, ser^15^, ser^20^, ser^37^, ser^46^, ser^392^ (20 h); ↓ acetyl-p53 @ Lys^373^ (5 h)	↑ p53 nucleus (20 h)	↑ 14-3-3o (5 h), ↑Gadd45a (5 h), ↓ Pgm (5 + 20 h), ↑ miR-34a (20 h)	n/a	Cell cycle regulation and senescence	Hypoxia

Notes: Literature (two studies) was reviewed and analyzed. Arrows indicate whether markers regulated by this form of stress increased (↑) or decreased (↓) in expression and/or activation. Various time measurements are indicated for the length of imposed stress. T.S. elegans: *Trachemys scripta elegans*.

Hypoxia, defined as ∼1% of physiological oxygen volume in skeletal muscle following an ischemic insult, represses myogenesis through numerous transcriptome changes [80,83]. One study found that hypoxia upregulated 641 genes involved in the cell cycle and in metabolism (HIF1-*α* and glycolysis), and downregulated 224 genes involved in protein catabolism and muscle organ development [80]. Of the specific genes upregulated, p53 expression was enhanced, with further augmentation of its downstream cell cycle, metabolic, and antioxidant signaling pathways to protect against oxidative-induced cell death [80,84].

It is well known that hypoxia inhibits myogenin and myogenic differentiation [81,85]. Recently, the mechanisms regulating this pathway have been elucidated and are dependent on p53 activation in a time-sensitive manner. Increased Ser^37^, Ser^46^, and Ser^392^ phosphorylation and decreased Lys^373^ acetylation occur at 5-h of anoxic exposure (complete oxygen deprivation), with additional phosphorylation at Ser^6^, Ser^9^, Ser^15^, and Ser^20^ at 20 h [84]. These post-translational modifications enhance p53 nuclear localization where it functions as a transcription factor of numerous negative regulators of myogenic differentiation, including those involved in cell cycle arrest, DNA repair, and apoptosis, which were observed to increase following anoxia [84]. Furthermore, p53 is necessary for the induction of the helix–loop–helix transcription factor Bhlhe40 which inhibits myogenin-driven terminal differentiation by reducing the binding of MyoD to the MyoG promoter [80]. Under hypoxic conditions specifically, p53 can upregulate Bhlhe40 expression to reduce the differentiation of myoblasts [80]. This repression of differentiation is additionally impacted by Notch activation, which translocates to the nucleus to reduce the activation of MyoD by creating interfering Hes1 and Hey1 heterodimers [85–87]. Repression is further accelerated by MyoD degradation through Bhlhe40 disruption of its binding to the myogenin promoter and through inhibition of the PI3K cell cycle progression and survival pathway [85,86,88,89]. Therefore, p53 plays a role in regulating the repression of myogenesis under hypoxic exposure. This may serve to allocate time towards upregulating stress–response genes involved in cell cycle repair and maintenance to enhance the prospect for cell survival and to transition the organism into hypometabolism with chronic oxygen deprivation.

### The role of p53 in mediating oxidative stress imposed by radiation

Ionizing radiation energy is an extreme stress signal which, depending on the length of exposure, can lead to cell death via two mechanisms. The first is through p53-dependent and -independent initiation of apoptosis which serves as a protective mechanism to eliminate heavily damaged or mutated cells [90]. The second mechanism is through the induction of irreversible G1/S cell cycle arrest which is dependent on ATM and p53, as well as its downstream target p21 [90,91]. Though the effect of radiation-induced death has been studied in many tissues, whether similar mechanisms are activated in skeletal muscle remains unknown. Further, the radiation dose and exposure time required to activate these signaling pathways in muscle tissue are under-discussed. Therefore two studies, measuring the effects of irradiation (5 Gy) exposure and its activation of the p53 signaling pathway were examined in this review (Table 6).

**Table 6.** Literature compiled on oxidative stress imposed through irradiation.

Publication information (Ref #)	Research model	Age at start (weeks)	Stress intensity	Length of stress	p53 expression (residue modification)	p53 cellular localization	Regulation of signaling pathways with stress		Signaling pathway(s) activated	Path model
							mRNA	Protein		
Yang et al., Cell Death, 2015 [92]	C2C12 myoblasts	n/a	Irradiation (5-Gy)	One-time; timecourse: 2, 6, 12, 24, 48, 72, 96 h post-IR	↑p53 (6 h)	↑ p53 nuclear (↑ myogenin – 2560 binding @ 6 h), ↓ 48 h (for MyHC, myogenin, H3K27Ac)	↓ Myogenin (48–96 h), ↓H3K27Ac	↓ MyHC	Myogenic repression, differentiation	n/a
Feng et al., Cancer Res, 2007 [95]	C57BL/6J p53 KO and WT *n*= 3	4–6	Irradiation (5 Gy)	8 and 24 h	No change	n/a	↑ TSC2, ↑ PTEN, ↑IGF-BP3, ↑p21, ↑Mdm2, ↑ Pirh2, ↑Cop1, ↑ CyclinG1, ↑Wip1 (8 + 24 h)	n/a	IGF-1-AKT-mTOR	Cancer cell growth

Notes: Literature (two studies) was reviewed and analyzed. Arrows indicate whether markers regulated by this form of stress increased (↑) or decreased (↓) in expression and/or activation. If a WT vs. KO model is employed, only the results from the WT group are indicated. Various time measurements are indicated for the length of imposed stress. KO: knockout; WT: wildtype; IR: irradiation; Gy: Gray.

The role of p53 on skeletal muscle differentiation following DNA damage is a field of interest because of its implications for the regenerative capacity of muscle. Previous work has revealed that p53 maintains the genetic stability of mouse embryonic stem (MES) cells following DNA damage by repressing Nanog, a cancer-stem cell marker, to then regulate and promote differentiation [92,93]. Regulation of MES cells is of key importance since skeletal muscle originates through myogenesis from the mesoderm, one of the three primary germ layers. Depending on the exposure time and intensity, p53 activation is required for cytokine-mediated inhibition of myogenic differentiation via TNF-*α*-driven apoptotic augmentation, leading to muscle atrophy [77,92,94]. Therefore, analysis of the regulation of key myogenic regulatory factors by p53 was examined under irradiation stress. The results indicate a direct role for p53 transcriptional repression of myogenin, with the likely purpose of ensuring adequate time for DNA damage repair and chromosomal segregation [92]. Furthermore, a time course was evaluated leading to the conclusion that a two-phase conditional regulation by p53 exists. Under non-ionizing radiation, p53 exerts low to minimal repression of the myogenin promoter, whereas under greater stress duration, p53 switches to become a negative regulator of myogenin to prevent aberrant cell proliferation [92].

Coordinated commitment to cell division is clearly monitored by p53-mediated intrinsic and extrinsic stress signals, such as DNA damage, hypoxia, low levels of ribosomal biogenesis, or oncogene activation, which introduces infidelity to cellular division through the inhibition of specific signals. Repression of such signals including the IGF-1-Akt and mTOR pathways allows for the coordinated activation of autophagy and apoptosis in a tissue-specific manner [95–98]. Ultraviolet radiation can slow ribosomal biogenesis and DNA damage, the effects of which lead to p53 activation and subsequent upregulation of downstream targets such as PTEN and IGF-BP3 [95,96]. These proteins function to shut down the IGF-1–AKT–mTOR signaling mechanisms in skeletal muscle to coordinate autophagy and apoptosis [95,96]. These results have implications for chronic physiological stress accumulating throughout a lifetime and indicate a role for persistent p53 repression of myogenin and cellular energy regulating pathways (IGF-1–Akt–mTOR) that may cumulatively lead to the development of myopathic diseases and aging overtime, and consequentially inducing muscle atrophy [92,95].

### The role of p53 in mediating oxidative stress imposed by chemical or medicinal agents

To further understand the activation of p53 and its downstream signaling pathways, various naturally occurring and synthetically manufactured drugs have been examined. These chemical agents, depending on whether they are direct or indirect oxidizing agents, trigger specific mechanisms to combat oxidative stress. Therefore, to determine how these oxidizing agents lead to variable signaling mechanisms, particularly stress signals activated by p53, a total of 11 studies (5 direct oxidizing agents and 6 indirect oxidizing agents) were analyzed (Table 7).

**Table 7.** Literature compiled on oxidative stress imposed through various types of chemical or medicinal oxidizing agents.

Publication information (Ref #)	Research model	Age at start (weeks)	Stress intensity	Length of stress	p53 expression (residue modification)	p53 cellular localization	Regulation of signaling pathways with stress		Signaling pathway(s) activated	Path model
							mRNA	Protein		
Direct oxidizing agents										
Pronsato et al., Steroids, 2016 [99]	C2C12 myoblasts	n/a	1 mM H_2_O_2_ ± 10^−9^ M TST	60 min TST + Timecourse H_2_O_2_: 4 h; 15 min, 30 min, 1–4 h	↑ p-p53 @ ser^15^ (1–2 h; TST ↓ p-p53	↑ Nuclear p-p53 (1–2 h)	↑ DNA fragmentation (70%), TST ↑ DNA fragmentation (44%) @ 4 h, ↑ p66Shc (1.5 h) TST ↓ p66shc)	↑ p-p66Shc @ ser^36^ (1–2 h) mito (TST ↓ p-p66shc), ↑ p-JNK1/2 @ Tyr^185^ + Thr^183^ (1–4 h) TST ↓ p-JNK), ↑p-PKC*β*1 @Thr^641^ (TST ↓ p-PKC*β*1)	Apoptosis	Sarcopenia
Hori et al., PLoS One, 2013 [105]	C2C12 myoblasts w/p53, SIRT1 KO	n/a	10 or 30 µM RSV for 3 hours + 50 µM AA or 50 µM H_2_O_2_	3 h RSV 24 h AA or H_2_O_2_	↑ acetyl-p53 @ Lys^379^ (AA) ↓ with RSV	n/a	n/a	↑caspase 3 (AA or H_2_O_2_)↓ w/ RSV, ↑ SOD2 (w/RSV), ↓ FOXO1/ FOXO3a/ FOXO4 (AA) ↓ acetyl-FOXO1 (w/ RSV), ↑Bax (AA ↓ w/ RSV, ↓ p-AMPK*α* @ Thr^172^ (AA), ↓p-ACC @ Ser^79^ (AA)	Apoptosis, antioxidant	n/a
Liu et al., Biochem Biophys Res Commun, 2007 [112]	C2C12 myoblasts with *α*B-crystallin OE, or control	n/a	0.5 mmol/L H_2_O_2_ in 10 ml	6, 12, 24 h	↑ p53 protein	↑p53/*α*B-crystallin binding cytoplasm (1 h); ↑ p53 mito	n/a	n/a	Apoptosis	n/a
Le Roux et al., Nat Commun, 2015 [110]	Tg:Pax7CT2 (Numb KO, p53 KO, Numb/p53 KO) w/ 4-OHT; satellite cells *n* = 3-4	n/a	20 µL (10 mg/ml) snake venom toxin injection or 10 mM cardiotoxin	Once; 5, 10, 21 DPI	↑ p53 protein (10DPI)	n/a	↓ Numb (5 DPI; ↑ 10 + 21 DPI)	↑Flk-1, ↑ p21 (10 DPI)	Muscle regeneration, cellular senescence	n/a
La Colla et al., J Cell Biol, 2017 [104]	C2C12 myoblasts	n/a	0.5 mM H_2_O_2_ ± 10^−8^ M E2	E2 1 h prior to H_2_O_2_; Timecourse: 30 min, 1, 3, 4 h	↑p-p53 protein @ ser^15^ (1–3 h); ↓ w/E2	n/a	↑Puma (1–4 h) ↓ w/E2, ↑ PERP (3 h) ↓ w/E2, ↓ Bcl-2 (3–4 h) ↑ w/E2, ↑ Bim (30 m–3 h) ↑ Mdm2 (30 min–1 h) ↑ w/E2	↑ p-FOXO3a @ser^253^ nuclear (30 m–1 h), ↑p-Akt (30 m–1 h), ↑ p-FoxO4 @ thr ^447–451^ (30 min–3 h) ↓ w/E2 @ 1 h	Differentiation, apoptosis	n/a
Indirect oxidizing agents										
Morimoto et al., J Atherscler Thromb, 2011 [67]	KK/Ay diabetic mice *n* = 5	9 weeks	2 mg/kg ATR	Once per day/4 weeks	↓ p53 protein	n/a	n/a	↑ p-IRS-1 @ tyr residues, ↑ p-Akt1 @ ser^473^, ↑p-Mdm2 @ ser^186^	Insulin resistance, proteasomal degradation	Type II diabetes
Shinozaki et al., Sci Signal, 2014 [73]	(1) F344 rats *n* = 7 (2) C2C12 myoblasts	(1) 100 weeks (2) n/a	(1)10 mg/kg 1400 W (2) 10 µg/ml LPS or 5 ng/ml TNF*α* or 50 ng/ml IFN-*γ* + 200 µM L-NIL or 50 µM 1400 W	(1) Once daily for 10 days (2) 72 h TNF-*α* or LPS or IFN-*γ*	(1) ↓acetyl-p53 @ Lys^379^ (2) ↑acetyl-p53 @ Lys^379^ by LPS, TNF-*α*, IFN-*γ* and 1400W (↓ L-NIL)	n/a	(1) n/a (2) ↑ Fas1, ↑ Tlr4, ↑ Murf1 by LPS, TNF-*α*, IFN-*γ* (↓ L-NIL)	(1) ↓S-NO SIRT1, ↓ acetyl-p65 @ Lys^310^; ↓ p65 (2)↑ S-NO SIRT1 by LPS, TNF-*α*, IFN-*γ* ↑acetyl- p65 @ Lys^310^ by LPS, TNF-*α*, IFN-*γ* (↓ L-NIL)	Inflammation, atrophy, nitrosative stress	Aging
Schwarzkopf et al., Genes Dev, 2006 [68]	C2 myoblasts	n/a	65 nM Dox ± 20 ng/ml TNF-*α*	24 h Dox, 8 h TNF-*α*	n/a	↑ Nuclear p53 and PW1	n/a	↓ Differentiation	Differentiation, regeneration	n/a
Aquilano et al., Antioxid Redox Signal, 2013 [48]	(1) C57/BL/6J and CDI mice *n* = 4 (2) C2C12 myoblasts	(1) 5 weeks (2) n/a	(1) 20 mM BSO, 4 mM L-NAME (2) 1 mM BSO, 0.1 mM L-NAME, 0.002 mM LY-83583	(1) 5 weeks oral (2) Once	(1) ↑ p53 protein (BSO)	(1) ↑ p53 binding to −2317 PGC-1*α* site (nuclear) (BSO) (2) ↑ p53 binding to −2317 PGC-1*α* site (nuclear) (BSO)	(1) ↑ PGC-1*α*, ↑ SOD2 (BSO) (2) ↑ PGC-1*α*, ↑ SOD2 (BSO) * all effects abolished by L-NAME	(1) ↓ GSH, ↑ PGC-1*α*, ↑ SOD2 (BSO) (2) ↓ GSH (6–48 h BSO), ↑ PGC-1*α* (BSO), ↑ SOD2 (BSO) * all effects abolished by L-NAME	Antioxidant	n/a
Baldelli and Ciriolo, Aging, 2016 [116]	C2C12 myoblasts	n/a	1 mM BSO 100 µM/ L-NAME	L-NAME (1 h prior BSO)	↑ S-NO p53 @ Cys^124^ (BSO)	↑ p53 at −2317 PGC-1*α* site (BSO) * all effects abolished by L-NAME	↑ PGC-1*α*, ↑ NFE2L2 and ↑ GCLC (BSO), ↑MyoD, Pax7 and Myogenin * all effects abolished by L-NAME	↑ PGC-1*α*, ↑ NFE2L2 and ↑ SOD2, ↑p21 (BSO)	Antioxidant, differentiation	n/a
Di et al., Mutagenesis, 2017 [121]	Mytilus galloprovincialis (mussels) – adductor muscle *n* = 6	n/a	56 µL/L B(*α*)P 1 mg/L C_60_	1 and 3 days ± 3 days recovery	↑ p53 mRNA (3 days B(*α*)P, (1–3 days C_60_), (1 day B(*α*)P + C_60_)	n/a	↑DNA breaks (1 + 3 days B(*α*)P ± C_60_) ↓ w/ recovery ↑ Ras (1 day B(*α*)P, 1 day B(*α*)P + C_60)_	↑ tGSH (3 days B(*α*)P or C_60_)	Atrophy, apoptosis	Environmental contaminants /carcinogens

Notes: Literature (11 studies) was grouped by direct oxidizing agents (5 studies) and indirect oxidizing agents (6 studies). Arrows indicate whether markers regulated by this form of stress increased (↑) or decreased (↓) in expression and/or activation. If a WT vs. KO model is employed, only the results from the WT group are indicated. Various time measurements are indicated for the length of imposed stress. Studies that perform experiments in both animal and cell culture models have the methodology and results numerically divided. KO: knockout), WT: wildtype; AA: antimycin A; DPI: days post-injury; ATR: Atorvastin; OE: overexpression; RSV: resveratrol; 4-OHT: 4-Hydroxytamoxifen; E2: 17*β*-estradiol; mito: mitochondria; Dox: doxorubicin; S-NO: S-Nitrosylation; TST: testosterone; LPS: lipopolysaccharide; BSO: Butathione Sulfoximine.

Direct oxidizing agents crucially impact metabolism and cellular homeostasis by acting as the final electron acceptor for many reactions. Numerous pharmacologic agents have been naturally discovered or synthetically constructed that fit the description of a direct oxidizing agent and consequentially play an important role in inducing or mediating oxidative stress through the activation of p53. Harmful oxidizing agents, such as H_2_O_2_, increase p53 expression which can activate kinases PKC*β* and JNK to phosphorylate p66Shc on its Ser^36^ residue, allowing for its translocation to the mitochondria [99–101]. Once in the mitochondria, p66Shc functions as a redox enzyme to amplify oxidative stress by provoking structural changes, such as mitochondrial outer membrane permeabilization, to further increase H_2_O_2_ generation and the release of cytochrome *c*[99,100]. p66Shc, when activated by H_2_O_2_, also inhibits the ERK signaling pathway, leading to inappropriate actin cytoskeleton polymerization and irregular glucose transport into skeletal muscle [99,102,103]. Under this form of oxidative stress, ERK is also known for abrogating the access of FOXO3a to DNA-binding sites by phosphorylating its threonine and serine residues [92,104]. Specific FOXOs that are activated with H_2_O_2_ include the JNK-mediated Thr^447^ and Thr^452^ phosphorylation of FOXO3a and FOXO4, respectively, which can then upregulate pro-apoptotic proteins Fas, TRAIL, and Bim [104–106]. Phosphorylation of p53 under H_2_O_2_ oxidative stress additionally leads to the transcription of pro-apoptotic proteins, including Bax, BH3-only family members, and PERP [99,104,107,108]. Antimycin A, another inducer of oxidative stress, functions by inhibiting the mitochondrial respiratory chain at complex III [105]. This activates alternative stress-resistant pathways through the SIRT1-mediated de-acetylation of p53 and concomitant FOXO activation [105,109]. Furthermore, this enhances the expression of ROS-detoxifying enzymes such as SOD2 and catalase [105,109]. Snake venom neotoxin/cardiotoxin, another direct oxidative stress inducer, elevates ROS to induce p38 MAPK-mediated Ser^33^ phosphorylation of p53 [110,111]. This leads to persistent senescence through Notch and Wnt signaling [88,110,111]. Interestingly, certain agents such as resveratrol, *α*-B-crystallin, E2 (17*β*-estradiol), and testosterone can protect against the H_2_O_2_-mediated p53 induction of apoptosis by enhancing the de-acetylation of p53 by SIRT1 or by binding certain pro-apoptotic family members [99,104,105,112]. p53 de-acetylation inhibits the transcription and subsequent synthesis of caspase 3 and Bax, and downregulates Puma and PERP which are upstream activators of Bax-dependent apoptosis [99,104,105,112]. This ultimately reduces ROS generation and p53-induced apoptosis.

Indirect oxidizing agents function through additional and alternate pathways to ultimately lead to similar results as direct oxidizing agents. Synthetic agents, in particular, have been created to mediate ROS production and regulate p53 signaling pathways depending on the pathology. For example, in diabetic hyperglycemia, p53 accumulates and activates downstream genes involved in myocyte death [113,114]. Desferrioxiamine, a hypoxia mimetic, leads to a similar accumulation of p53 as seen with diabetes, ultimately to activate apoptosis [67]. Atorvastatin, on the other hand, is a medicinal statin used to treat diabetes and high cholesterol, and serves to reduce p53 accumulation by increasing IRS-1/Akt phosphorylation of Mdm2 to upregulate p53 ubiquitination and degradation [67,115].

Specific indirect oxidizing agents, such as PTIO, LY-83583, and buthionine sulfoximine, induce oxidative stress through the upregulation of NO/iNOS/nNOS and/or GSH depletion pathways [48,73,116]. As a first response compensatory mechanism, the antioxidant pathway is upregulated to control this redox imbalance before stress can consume the cell and initiate apoptosis. The antioxidant defence system is activated by the inflammatory cascade initiated by GSH decrements and increased NO/cGMP which leads to the activation of p53 [48,73,116]. Activated p53 is a result of either increased inhibition of its negative regulators Mdm2 and SIRT1, or through Cys^124^ S-nitrosylation of p53 due to NO flux, allowing it to localize to the nucleus [48,73,116]. Once in the nucleus, p53 binds to the PGC-1*α* promoter to enhance its transcription [48,116,117]. Both p53 and PGC-1*α* then act synergistically to co-activate the Nrf2-mediated antioxidant response to buffer harmful ROS/RNS [48,117]. However, as stress progresses and cannot be alleviated by increased antioxidant production, pre-apoptotic pathways such as the inflammation pathway, are activated. Specifically, the p53 gene target PW1 physically interacts with TRAF2 to activate the cytokine TNF-*α*–NF-kB pathway under chemically induced oxidative stress [68,118]. This provokes protein degradation and prevents muscle differentiation by upregulating the ubiquitin-proteasome pathway via atrogin-1, and additionally initiating apoptosis by enhancing bax mitochondrial localization [68,78,119]. Depending on the length of chemical activation, chronic exposure can lead to SIRT1-mediated de-acetylation of PGC-1*α*, p53-mediated regulation of damage associated molecular patterns (DAMPs), or increased binding between p53 and MURF1 to trigger ubiquitination [48,73]. These changes ultimately lead to progressive inflammation, premature atrophy, and cell death [48,73,116,120]. For example, chronic exposure to environmental contaminants such as polycyclic aromatic hydrocarbon benzo(*α*)pyrene and C_60_ fullerene pollutants act to initially increase antioxidant defences by increasing glutathione levels [121,122]. However, as the oxidative stress consumes these defences, these pollutants switch to act as genotoxins by producing hydroxyl and superoxide anion radicals that react with DNA, triggering DNA strand breaks which then activate p53-mediated induction of apoptosis [121,122]. Therefore, both direct and indirect oxidizing chemicals provide insight on the p53-mediated regulation of low to high levels of oxidative stress, and elucidate some of the mechanisms activated with various drugs.

## Summary and conclusion of cited studies

**Figure 2.**
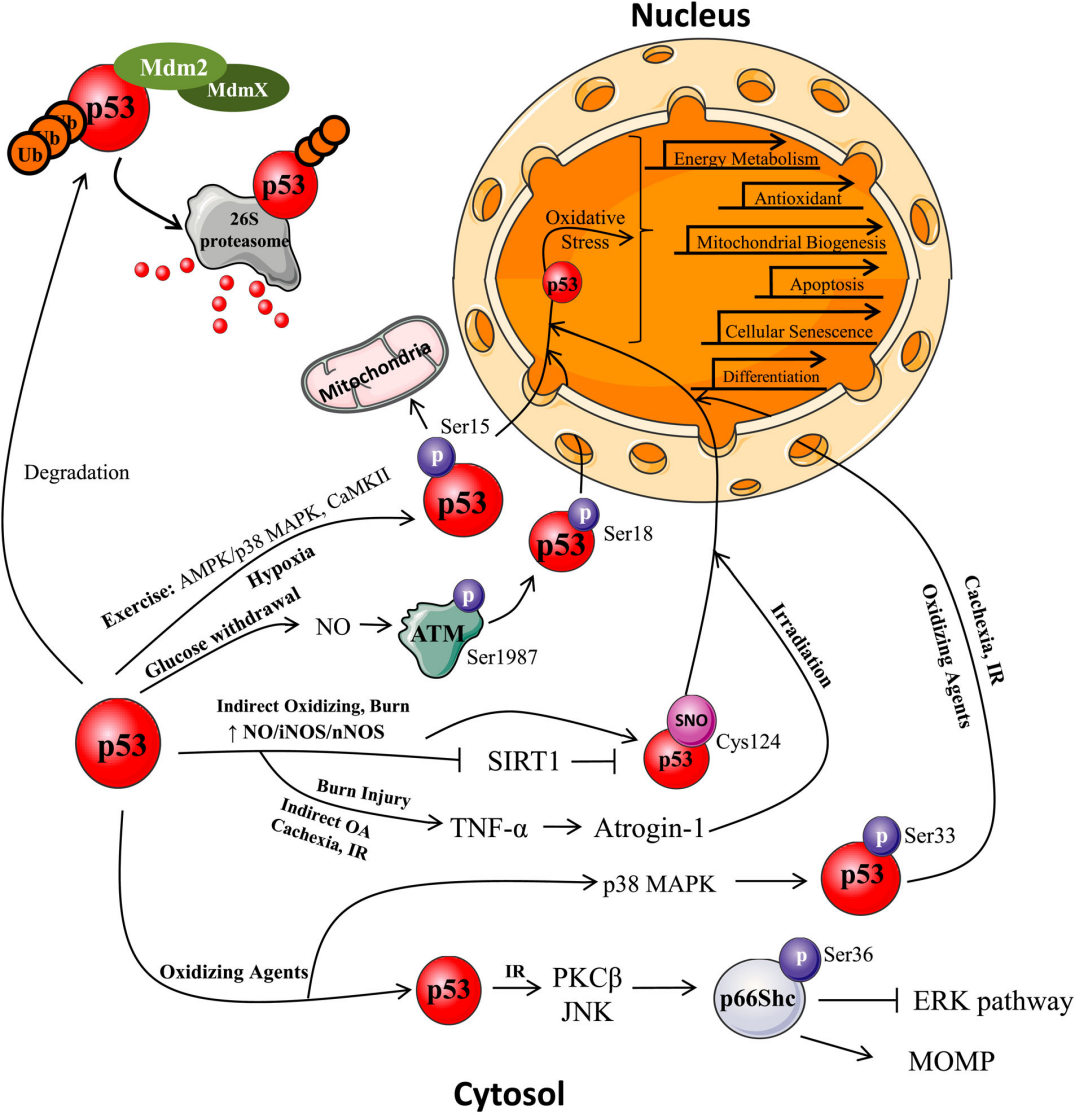
p53 subcellular localization induced by oxidative stressors. p53 is normally maintained at low levels in the cytosol by its negative regulator Mdm2. When Mdm2 dimerizes, p53 is poly-ubiquitinated and targeted for degradation by the 26S proteasome [67,115]. With the induction of stress, p53 post-translational modifications allow for its subcellular redistribution. *Exercise.* Muscular contractions induce the activation of numerous signals, such as kinase phosphorylation, increased ROS production, enhanced cytosolic calcium concentration, and an increased AMP: ATP ratio, ultimately leading to p53 Ser15 phosphorylation [12,17,22]. Once activated, p53 localizes to the nucleus to increase NUGEMP transcription, and to the mitochondria to regulate mtDNA transcription. p53 additionally regulates energy metabolism and DNA repair pathways [26,32,36,37,42,52]. *Diet modification*. With caloric restriction/glucose withdrawal there is an upregulation of NO which activates ATM kinase to phosphorylate p53 (Ser18) leading to its nuclear localization [38,49]. In the nucleus, p53 regulates energy metabolism, antioxidant, cellular senescent, and apoptotic pathways [38,44,47–50,53,54]. *Tissue manipulation*. With immobilization and cachexia-mediated atrophy, there is p53-mediated transcriptional activation and repression of cell cycle arrest and myogenic genes, respectively [68,75]. With burn injuries, there is an increase in cytokine-mediated activation of iNOS to produce NO which inhibits SIRT1 leading to the activation of p53 as well as increased TNF-*α* and atrogin 1 expression to induce muscle wasting [68,71,77,78]. *Oxygen deprivation*. Acute and prolonged hypoxia induce p53 phosphorylation at numerous sites to enhance the repression of myogenic differentiation and upregulate cell cycle arrest and apoptotic pathways [80,84,86–88]. *Irradiation*. Under IR stress, TNF-*α* driven apoptosis is induced through p53 activation, to enhance nuclear apoptotic signaling pathways in addition to increasing p66Shc phosphorylation to initiate MOMP [92,101]. *Direct/indirect oxidizing agents*. Oxidizing agents such as H_2_O_2_ lead to the activation of cell death pathways mediated by phosphorylated p53 to initiate persistent senescence and apoptosis [107,108,111]. Furthermore, p53 activates kinases to phosphorylate p66Shc, inhibit ERK signaling, prevent cytoskeleton polymerization, and to induce MOMP [99–101,103]. With indirect oxidizing agents, NO/iNOS/nNOS pathways are upregulated to activate p53 by reducing the inhibition by its negative regulator SIRT1 and by S-nitrosylation of p53. This enhances nuclear localization for antioxidant and apoptotic transcription with increasing stress, and increases atrogin-1 mediated ubiquitin-proteasome activation [48,73,78,116,117].

Oxidative stress in skeletal muscle is a result of impaired redox homeostasis due to either the overproduction of ROS, or a compromised antioxidant defence system. ROS overproduction, caused by either endogenous or exogenous sources, can lead to damaged proteins, lipids, and DNA, inevitably causing pathologies such as metabolic (type II diabetes and cancer), neuromuscular (dystrophies), neural (Alzheimer’s disease) or cardiovascular diseases, as well as promoting muscle aging [123,124]. Endogenous sources of ROS, such as those produced by exercise, as well as exogenous sources, such as excess nutrient availability, radiation exposure, hypoxia, and tissue manipulations, can lead to p53 subcellular localization for the regulation of specific signaling pathways to alleviate the imposed oxidative stress and restore cellular homeostasis (Figure 2).

Depending on the type, duration, and intensity of the imposed stress, various signaling pathways are activated within specific organelles, such as the mitochondria or nucleus, to mitigate the cellular damage caused by these non-physiological levels of ROS. Specifically, p53 is deemed to be of key importance in regulating these cellular stress responses. The studies outlined in this review confirm a dual ability for p53 activation of specific signaling mechanisms, dependent on the intensity and length of the oxidative stress. With low levels of oxidative stress induced by dietary restriction, non-ionizing radiation, hypoxia, and oxidizing agents, there is an upregulation of p53-mediated activation of the antioxidant enzymes Nrf2, SOD2, sestrins, and catalase. Furthermore, cell cycle arrest and cellular senescent proteins p21 and Gadd45a are upregulated, concomitant with increased autophagy to maintain genomic integrity as well as a healthy organelle pool, respectively. Interestingly, metabolic modifications additionally occur exemplified by increased TIGAR, PGM, and GLS2 expression which reduce glycolysis and pyruvate levels, while increasing Lipin-1 to upregulate FAO. Surprisingly, with exercise, p53 enhances cellular function by increasing mitochondrial biogenesis and maintaining mtDNA utility.

Yet with greater and prolonged levels of stress, signaling pathways that exacerbate oxidative dysfunction, such as those involved in inflammatory cascades and cell death, are intensified. As observed with chronic nutrient abundance, anoxia/hypoxia, and burn injuries, there is increased expression of inflammatory proteins and cytokines including c-myc, TNF-*α*, IL-1*β*, Bhlhe4, and Notch. Immobilization of muscle on the other hand, a mechanism solely dependent on duration, is characterized by increased ATF4, p21, and atrogin-1 expression to cause tissue atrophy. With even greater levels of stress induced by radiation and chronic exposure to oxidizing agents, there is an increase in numerous signaling pathways including TNF-*α*-PWI-caspase-Bax, PTEN, FOXOs, p66Shc, MURF1, and IGF-BP3, which initiate the accumulation of harmful free radical species leading to cell death. In these cases, p53 acts as a pro-oxidant to further exacerbate the stress load. This culminates in the inhibition of preventative oxidative signaling pathways and enhances the caspase cascade to initiate apoptosis. Interestingly, this duality function of p53 is observed with specific oxidative stress sources such as radiation, nutrient regulation, hypoxia, and oxidizing/non-oxidizing agents, but not with exercise and tissue manipulation.

**Figure 3.**
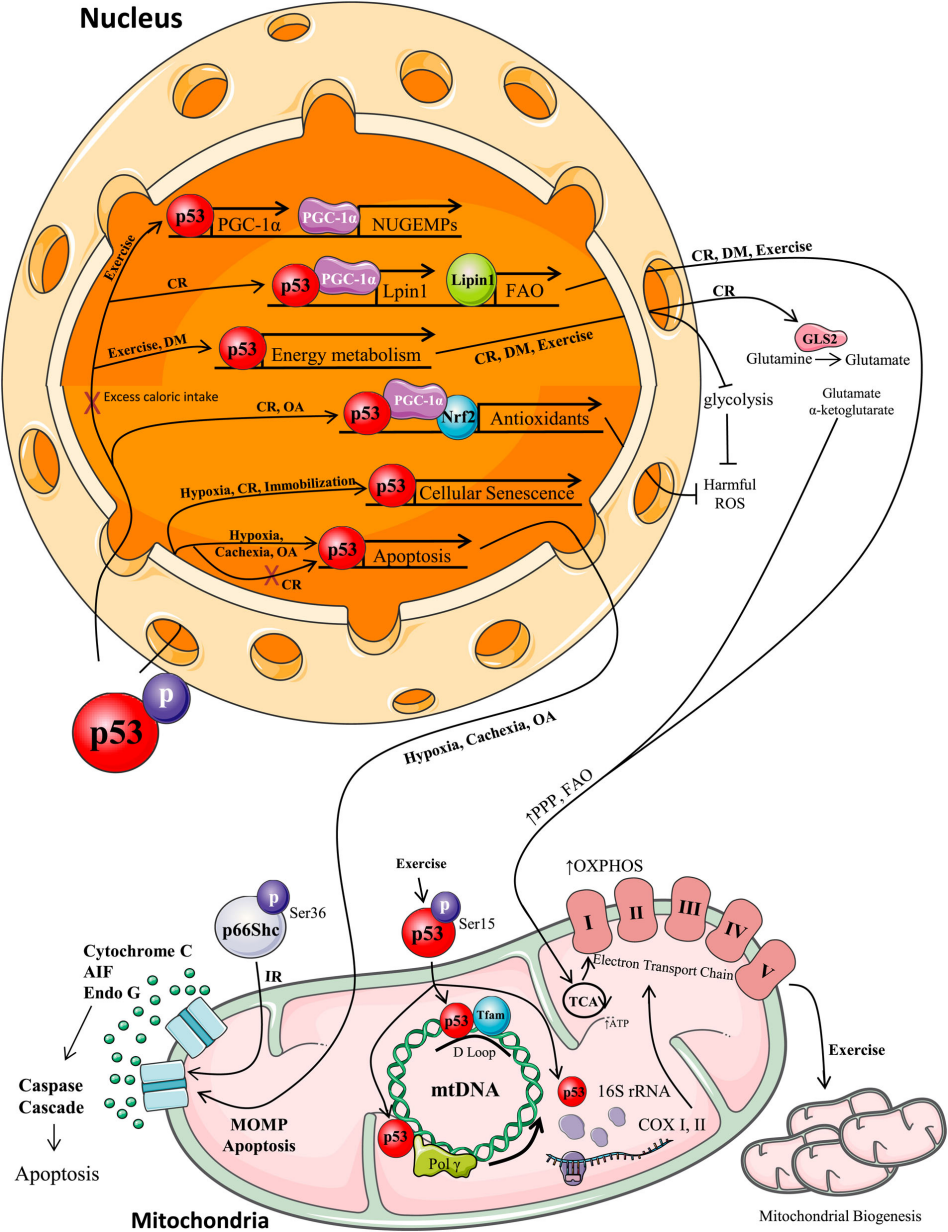
p53 nuclear and mitochondrial localization and regulation of signaling mechanisms with oxidative stress. With the induction of stress, p53 post-translational modifications allow it to localize to various cellular compartments to regulate cellular homeostasis. *Exercise*. Once phosphorylated, p53 localizes to the mitochondria to assist Tfam and Pol*γ* binding to mtDNA, and to increase 16S rRNA production to enhance mitochondrial biogenesis [12,17,24,26,29,30]. p53 also translocates to the nucleus to increase PGC-1*α* expression and activate NUGEMPs transcription [12,17,22–26,48]. Furthermore, p53 can regulate glycolysis by reducing glycolytic energy production and enhancing the pentose phosphate, fatty acid oxidation, and oxidative phosphorylation energy pathways [26,36,37,42,52]. *Diet Modification*. With diet modifications (DM), there is a p53-mediated upregulation of energy metabolism genes to reduce glycolysis and upregulate oxidative phosphorylation [36,37]. With caloric restriction (CR) or glucose withdrawal, p53 co-localizes in the nucleus with PGC-1*α* to enhance Lipin-1 expression for increased FAO. p53 upregulates enzymes for mitochondrial substrate provision, increases antioxidant defenses, increases cellular senescent and cell cycle arrest genes, and decreases apoptotic gene transcription [38,44,47–50,53,54]. On the other hand, excess caloric intake leads to p53 transcriptional repression (exemplified by red X) of GLUT 1 and GLUT 4 genes, reduces PGC-1*α* and NUGEMP activation, and impairs FAO gene transcription, leading to impaired energy utilization and fat accumulation [56–59,61]. *Tissue Manipulation*. With immobilization there is an p53-mediated transcription of cell cycle arrest genes as well as an increase in Id2 to decrease DNA-binding and transcriptional activity [68,75]. *Oxygen Deprivation*. Hypoxia induces p53 phosphorylation at numerous sites leading to cell cycle arrest and apoptotic transcriptional activation. *Irradiation*. Under IR stress, p53 increases p66Shc phosphorylation, to coordinate autophagy and apoptosis, in order to regulate cellular integrity [101,103]. *Direct/Indirect Oxidizing Agents*. Oxidizing agents, such as H_2_O_2_, lead to the activation of cell death pathways mediated by phosphorylated p53 to initiate persistent senescence and to enhance transcription of pro-apoptotic proteins such as Bax and PERP [107,108,110,111]. With indirect oxidizing agents, p53 nuclear localization increases antioxidant transcription. With prolonged exposure apoptosis can be induced [48].

This review outlines for the first time the various modalities of imposed oxidative stress in skeletal muscle that analyze the regulation of p53 and its numerous downstream signaling pathways involved in maintaining cellular homeostasis (Figure 3). Clearly the integration of p53 into regulatory networks that combat oxidative stress has allowed this protein to earn its title as the ‘Guardian of the Genome’. This exciting avenue of research concerning the regulation of oxidative stress by varying mechanisms activated by p53 will significantly contribute to existing knowledge on how p53 can be therapeutically targeted to treat numerous disorders and diseases for which oxidative stress is a major contributory factor.

## Appendix Appendix

**Table A.** Human studies excluded from systematic analysis and compilation in the data tables. Throughout the study, human models were largely excluded to enable a greater focus on the role of p53 in animal skeletal muscle and cell culture models. A list has been created including relevant primary human research on p53 in skeletal muscle. Search criteria include ‘p53’ AND ‘human’ AND ‘skeletal muscle’ between January 1, 1990 and March 1, 2017. This is compiled for the purpose of further viewing and analysis by academic audiences.

Title	Authors	Year	Journal
Postexercise high-fat feeding suppresses p70S6K1 activity in human skeletal muscle	Hammond KM, Impey SG, Currell K, et al.	2016	Med Sci Sports Exerc
Mitochondrial adaptations to high-volume exercise training are rapidly reversed after a reduction in training volume in human skeletal muscle	Granata C, Oliveira RS, Little JP, et al.	2016	FASEB J
Acute endurance exercise induces nuclear p53 abundance in human skeletal muscle	Tachtsis B, Smiles WJ, Lane SC, et al.	2016	Front Physiol
1*α*,25-dihydroxyvitamin D3 regulates mitochondrial oxygen consumption and dynamics in human skeletal muscle cells	Ryan ZC, Craig TA, Folmes CD, et al.	2016	J Biol Chem
Training intensity modulates changes in PGC-1*α* and p53 protein content and mitochondrial respiration, but not markers of mitochondrial content in human skeletal muscle	Granata C, Oliveira RS, Little JP, et al.	2016	FASEB J
Differential expression of perilipin 2 and 5 in human skeletal muscle during aging and their association with atrophy-related genes.	Conte M, Vasuri F, Bertaggia E, et al.	2015	Biogerontology
An examination of resveratrol’s mechanisms of action in human tissue: impact of a single dose in vivo and dose responses in skeletal muscle *ex vivo*	Williams CB, Hughes MC, Edgett BA, et al.	2014	PLoS One
Transcriptomic profiling of TK2 deficient human skeletal muscle suggests a role for the p53signalling pathway and identifies growth and differentiation factor-15 as a potential novel biomarker for mitochondrial myopathies	Kalko SG, Paco S, Jou C, et al.	2014	BMC Genomics
Increased Plin2 expression in human skeletal muscle is associated with sarcopenia and muscle weakness	Conte M, Vasuri F, Trisolino G, et al.	2013	PLoS One
Reduced carbohydrate availability enhances exercise-induced p53 signaling in human skeletal muscle: implications for mitochondrial biogenesis	Bartlett JD, Louhelainen J, Iqbal Z, et al.	2013	Am J Physiol Regul Integr Comp Physiol
The effect of resistance exercise on p53, caspase-9, and caspase-3 in trained and untrained men	Sharafi H and Rahimi R.	2012	J Strength Cond Res
Matched work high-intensity interval and continuous running induce similar increases in PGC-1*α* mRNA, AMPK, p38, and p53 phosphorylation in human skeletal muscle	Bartlett JD, Hwa Joo C, Jeong TS, et al.	2012	J Appl Physiol (1985)
Regenerative potential of human muscle stem cells in chronic inflammation	Duijnisveld BJ, Bigot A, Beenakker KG, et al.	2011	Arthritis Res Ther
